# Exploring the relationships between neuronal parameters and network activity

**DOI:** 10.1186/1471-2202-13-S1-P172

**Published:** 2012-07-16

**Authors:** Anca Doloc-Mihu, Ronald L Calabrese

**Affiliations:** 1 Department of Biology, Emory University, Atlanta, GA 30322, USA

## 

This study constitutes our next step toward a full investigation of how intrinsic membrane and synaptic parameters affect the electrical activity of a half-center oscillator (HCO) model and how different parameter regimes influence stability and modulatability of the HCO model’s output. In our previous study [3], we constructed an efficient relational database table with the resulting characteristics of Hill et al.’s [2] HCO single-compartment conductance-based model. This model consists of two reciprocally inhibitory neurons and replicates the electrical activity of the oscillator interneurons of the leech heartbeat CPG under a variety of experimental conditions.

We systematically [4] explored the parameter space of this HCO model by varying a set of eight selected parameters (maximal conductances of intrinsic and synaptic currents) in all combinations possible, resulting in 10,485,760 simulated model instances (10,321,920 HCO instances and 163,840 corresponding isolated neuron instances). We classified within a group the simulated instances showing similar electrical activity. The HCOs were split into ten groups labeled: ***spiking***, ***silent***, ***asymmetric activity***, ***plateau*, *irregular spikes*, *asymmetric bursting*, *one burst*, *irregular period*, *unbalanced***, and ***functional*** (1,202,139 instances); the isolated neuron instances were split into seven groups: ***spiking***, ***silent***, ***bistable***, ***plateau***, ***irregular spikes***, ***irregular period***, and ***regular bursting ***[3].

By querying the database we started analyzing the relationships between the neuronal parameters of the model instances within the same group and across groups. Model instances whose neurons are synaptically isolated are called isolated neuron instances. We found that ***regularly bursting*** (isolated) neuron instances compose only a small minority of ***functional*** HCOs (424 instances); the vast majority was composed of ***spiking*** neurons (32,568 instances). Also, our data suggest that ***regular bursting*** isolated neurons form ***functional*** HCOs that are more robust to variations in synaptic parameters than those formed by ***spiking*** isolated neurons. Approximately 8.2% (99,066 instances) of the functional HCOs have bursting characteristics that are similar to those of the living animal [1].

We used dimensional stacking (the NDVis tool) to visualize the distribution of each group of model instances versus the neuronal parameters. While each group has its own distribution within the parameter space, the groups of bistable and regular bursting isolated neuron instances separate large zones of spiking and silent model instances.

To explore in depth the interaction of parameters that lead to the different activity groups we have identified, we used the Principal Component Analysis (PCA) method. When we applied the PCA to the group of regular bursting isolated neuron instances, we found that only 4 (out of 6) components are important for this group (sum of variances > 95%) and that the 1^st^ component has the greatest impact (variance > 60%). This component is mostly influenced by the maximal conductances of NaP, K_2_ and Leak, and the E_Leak_ (linear combination of the parameters). Then, we applied PCA to our group of functional HCOs showing realistic leech bursting characteristics and we found that the first 6 (out of 8) are the most important principal components for this group (sum of variances > 96%). However, unlike in the regular bursting group, none of these principal components is dominant. This suggests that this group has complex non-linear relationships between its parameters and to explore them we need to use more sophisticate computational methods that the (linear) PCA.

## References

[B1] Cymbalyuk GS Gaudry Q Masino MA Calabrese RL Bursting in leech heart interneurons: cell-autonomous and network-based mechanisms J Neurosci 2002 22 10580 10592 12486150 10.1523/JNEUROSCI.22-24-10580.2002 PMC6758431

[B2] Hill AAV Lu J Masino MA Olsen H Calabrese RL A model of a segmental oscillator in the leech heartbeat neuronal network J Comp. Neurosci 2001 10 281 302 10.1023/A:1011216131638 11443286

[B3] Doloc-Mihu A Calabrese RL Bringing up the rear: new premotor interneurons add regional complexity to a segmentally distributed motor pattern J Biol Physics 2011 37 263 283 10.1007/s10867-011-9215-y PMC3214089 21775711

[B4] Prinz AA Billimoria CP Marder E Alternative to hand-tuning conductance-based models: construction and analysis of databases of model neurons J Neurophysiol 2003 90 3998 4015 10.1152/jn.00641.2003 12944532

